# Ethnobotany of wild edible plants in multiethnic areas of the Gansu–Ningxia–Inner Mongolia junction zone

**DOI:** 10.1186/s13002-022-00549-1

**Published:** 2022-08-09

**Authors:** Xiaohuan Jia, Yongxia Zhao, Yunyue Zhu, Xin Zeng, Xuehui Liang, Jian Xie, Faming Wu

**Affiliations:** 1 School of Pharmacy, Zunyi Medical University, Zunyi, 563000 China; 2 School of Preclinical Medicine, Zunyi Medical University, Zunyi, 563000 China; 3 Jingyuan County Water Affairs Bureau, Baiyin, 730900 China; 4 College of Pharmacy, Zunyi Medical University, Zunyi, 563000 China

**Keywords:** Ethnobotany, Wild plants, Healthy plants

## Abstract

**Introduction:**

In recent years, research on wild edible plant resources has become increasingly popular. The Hassan Nature Reserve is a multiethnic area mainly composed of people belonging to the Han, Hui, and Mongolian groups. The utilization of edible wild plant resources in this area is extremely high. However, with the advancement of urbanization and the development of modern agricultural technology, these resources have been seriously damaged, and related traditional knowledge, such as that related to national medicine, has been lost.

**Methods:**

Based on a literature study, interviews with village and community organizations, participatory observation, and quantitative evaluation of ethnobotanical resources, wild edible plants in the Gansu–Ningxia–Inner Mongolia junction zone, were investigated.

**Results:**

The survey results showed that there were 53 species (varieties) of wild edible plants belonging to 24 families in this area. The Compositae and Liliaceae families were the most abundant, with 8 and 7 species, respectively. The young stems and leaves were the most edible parts of the plants, as observed for 17 species, followed by fruits (including young fruits), which were considered the edible part of 16 species. Other edible parts included the roots or rhizomes (bulbs), seeds, whole plants, skins, etc. The edible plants were consumed in two forms: raw and cooked; raw plants, mainly fruit, were typically consumed as snacks. The cooked foods mainly consisted of vegetables, with tender stems and leaves as the main food source. These components were also used as seasoning, in medicinal diets, and as an emergency food source in times of famine. Important (CFSI > 500) wild edible plants used in health care in the region include *Mulgedium tataricum* (L.) DC., *Nostoc commune* Vaucher ex Bornet & Flahault, *Sonchus arvensis* L., *Taraxacum mongolicum* Hand.-Mazz., *Allium schoenoprasum* L., *Robinia pseudoacacia* L., *Hemerocallis citrina* Baroni, *Elaeagnus angustifolia* L., *Medicago sativa* L., *Ulmus pumila* L., *Stachys sieboldii* Miq., and *Toona sinensis* (Juss.) M. Roem., and these plants had high utilization values and rates locally.

**Conclusion:**

In summary, the species of wild edible plants and their edible parts, categories, consumption forms and roles in health care in this area are diverse. The utilization of traditional knowledge is rich, and some wild plants have high development value.

## Background

Wild edible plants have played a very important role in the history of human development [1], especially in times of famine. Wild edible plants provide not only life-saving foods but also life-saving medicines [2]. By providing abundant materials, wild edible plants are also an indispensable part of people's lives [3]. Because wild edible plants are derived from natural resources and play important roles in health care, they are respected in modern societies and have great market potential [4, 5]. Research on wild edible plant resources has been carried out both in China and abroad. In particular, plant resources have become a hot research topic in recent years. Many products derived from wild resource have become valued goods in the market and popular health foods [6–10]. Scholars in China have mainly focused on the investigation and study of rare or uncommon edible wild plant resources [11–14], and these studies have provided informational resources for the development and utilization of local wild edible plant resources.

The Hassan Nature Reserve is located in the arid area of the Loess Plateau, where Gansu, Ningxia and Inner Mongolia meet. The region is characterized by multiethnic groups, mainly composed of Han, Hui, and Mongolian people. Due to the relatively low precipitation and high evaporation in this area [15], plant resources are relatively scarce, both in the number of species and in abundance. Since ancient times, this has been a poverty-stricken area with a shortage of materials. However, exchanges and integration among the Hui, Han, and Mongolian ethnic groups, each of which has a long history with accumulated traditional experience with wild edible plants, has contributed to the extremely high degree of exploitation of the edible wild plant resources in the region. However, with the advancement of urbanization and the development of modern agricultural technology, such resources have been seriously damaged, and related traditional knowledge, such as indigenous medicine, has been lost. Therefore, this study explored, recorded, and summarized the traditional knowledge of edible wild plant resources in this area. This knowledge has been very beneficial in the protection of traditional cultures and the sustainable development and utilization of wild edible plant resources.

## Methods

### Study area

**Fig. 1:**
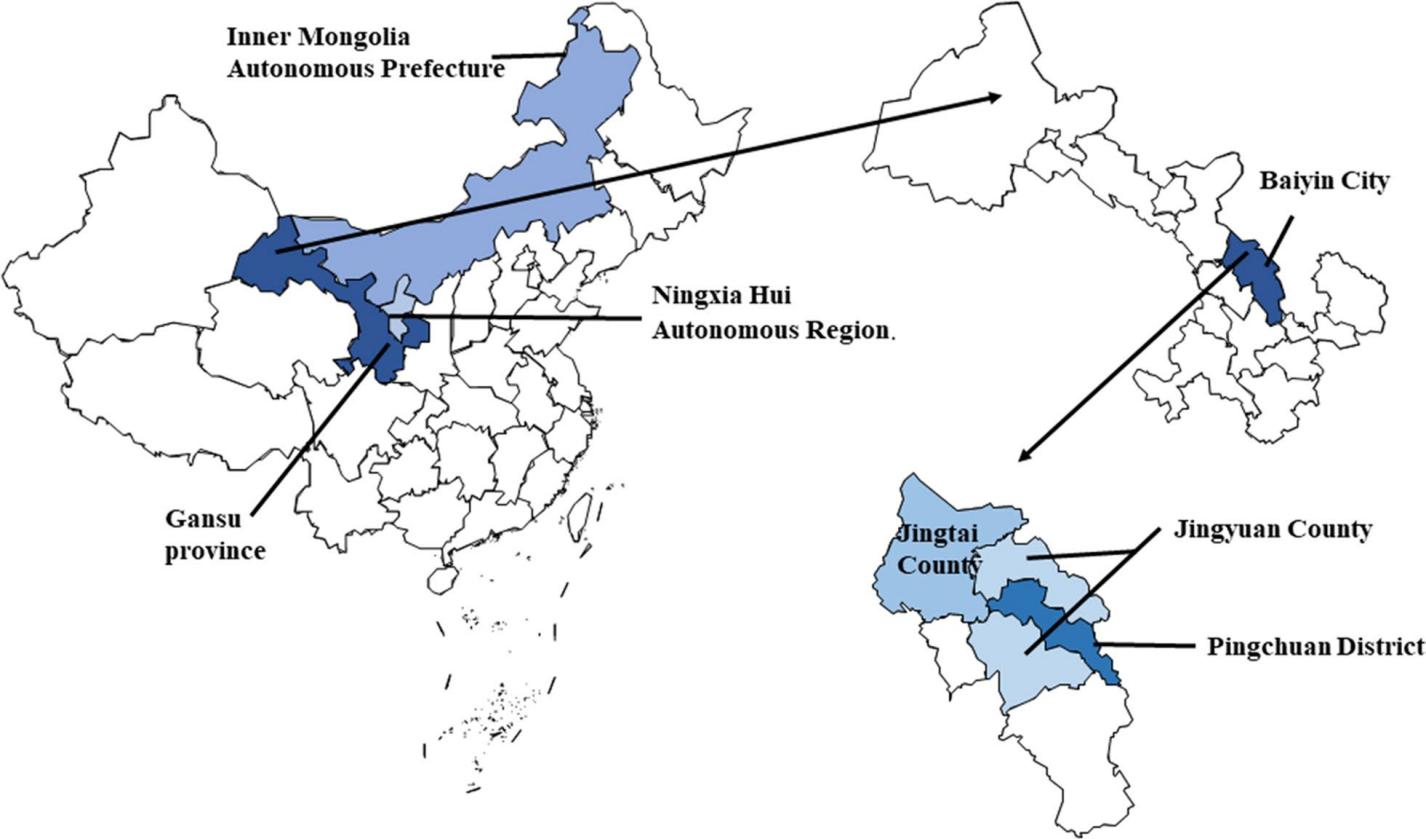
Survey area

In this study, Jingyuan County, Pingchuan district, and Jingtai County in Baiyin city, Gansu Province (Fig. 1), with Hassan Mountain Nature Reserve as the center point, were investigated. The area spans the latitudes of 36 to 37 38' north and longitudes of 103 33' to 105 51' east. It is located in the transition zone of three major regions: the Loess Plateau in western Gansu Province, the Yanyu region of the Qilian Mountains in Shandong Province, and the Tengger Desert. It occurs in a transition zone, spanning semiarid, middle temperate, and arid zones. The annual average temperature is 6–9 °C, and the annual rainfall is 180–450 mm [16]. The area is home to multiple ethnic groups, and the main ethnic groups in the territory are the Han, Hui, and Mongolian. Additional ethnic groups in the area include the Tibetan, Manchu, and other ethnic groups [17]. The economy of the area is based on agriculture and industry, and the main crops are *Triticum aestivum* L., *Zea mays* L., *Oryza sativa* L., *Chenopodium quinoa* Willd., *Solanum tuberosum* L., *Vicia faba* L., *Linum usitatissimum* L., *Allium cepa* L., *Brassica rapa* L., *Ziziphus jujuba* Mill., *Lycium chinense* Mill., etc. [18–20]. In addition, the region is also an important post-station along the Silk Road; relying on the advantages provided by this resource, it is active in the development of characteristic industries and integrates multiple cultures, such as the Yellow River culture, the Silk Road culture, the farming culture, the folk culture, and the red culture, forming a unique local culture [21].

### Ethnobotanical information collection

During the field investigation, interviews with key individuals, semistructured interviews, and participatory rural evaluation methods were used. The basic format of the interviews followed the “5 W + 1H” structure [22] to cover the traditional knowledge of edible wild plants; in addition, basic information about the interviewee as well as the local common names, edible parts, categories, consumption methods, and health care functions of edible plants were recorded, organized, and analyzed. The participatory observation method [23] was also used to understand the types, uses, functions, edible parts, and consumption methods of wild plants collected and eaten by the local people in their daily life. Interviews were also conducted by telephone communication, and the format of these interview was the same as that of the field interviews.

### Quantitative evaluation of ethnobotanical resources

The national plant cultural importance index (CFSI) was used to evaluate the wild edible plants in the investigated area.

$$\begin{aligned} {\text{CFSI}} = & {\text{FQI}} \times {\text{AI}} \times {\text{FUI}} \times {\text{PUI}} \\ \times & {\text{MFFI}} \times {\text{TSAI}} \times {\text{FMRI}} \times 10^{{ - 2}} \\ \end{aligned}$$

where FQI is the frequency index, AI is the commonness index, FUI is the frequency index, PUI is the utilization site index, MFFI is the multifunctional food utilization index, TSAI is the taste evaluation index, and FMRI is the medicinal diet function index [24].

Each index was graded and assigned a value according to the Research Methods of Ethnobotany [25]. The frequency of quotation index (FQI) is the number of people who mentioned a plant in all the informational reports. The availability index (AI) was divided into very common (4.0), common (3.0), general (2.0), and uncommon (1.0). The frequency of utilization index (FUI) was divided into more than once a week (5.0), once a week (4.0), once a month (3.0), more than once a year but less than once a month (2.0), once a year (1.0), and not used for nearly 30 years (0.5). The parts used index (PUI) was divided into whole plant (4.00), aboveground and belowground parts (3.00), tender leaves and tender stems and leaves (2.00), flowers, fruits (1.50), tender roots, stems, and stipules (1.00), and tender buds (0.75). The multifunctional food use index (MFFI) was divided into raw food and cold mix (1.5), boiling, stewing, and flavoring (1.0), special-purpose flavoring (0.75), and raw food as snack (0.50). The taste score evaluation index (TSAI) was used to classify plants as excellent (10.0), very good (9.0), good (7.5), average (6.5), poor (5.5), and very poor (4.5). The food and medicinal role index (FMRI) was divided into very high (5.0 as medicine and food), high (4.0 for treating certain diseases as medicine), medium–high (3.0 for very healthy food), medium–low (2.0 for healthy food, efficacy unknown), and unknown (1.0).

### Specimen identification

The collected plants were identified to the species level by referencing the full-text electronic edition of Flora of China (http://www.iplant.cn/frps) [26], Atlas of Desert Plants in China [27], and Field Identification Manual of Common Plants in China (Qilian Mountain Volume) [28]. Various information materials were collected and analyzed for this research, and representative samples were drawn. Herbarium specimens are stored at the Life Science Museum of Zunyi Medical University (No.6 Xuefu West Road, Honghuagang district, Zunyi city, Guizhou Province, China).

The research was carried out following the code of ethics of the American Anthropological Association [29] and the International Society of Ethnobiology Code of Ethics [30]. Oral prior informed consent was acquired.

## Results

### Basic information from reports

**Fig. 2:**
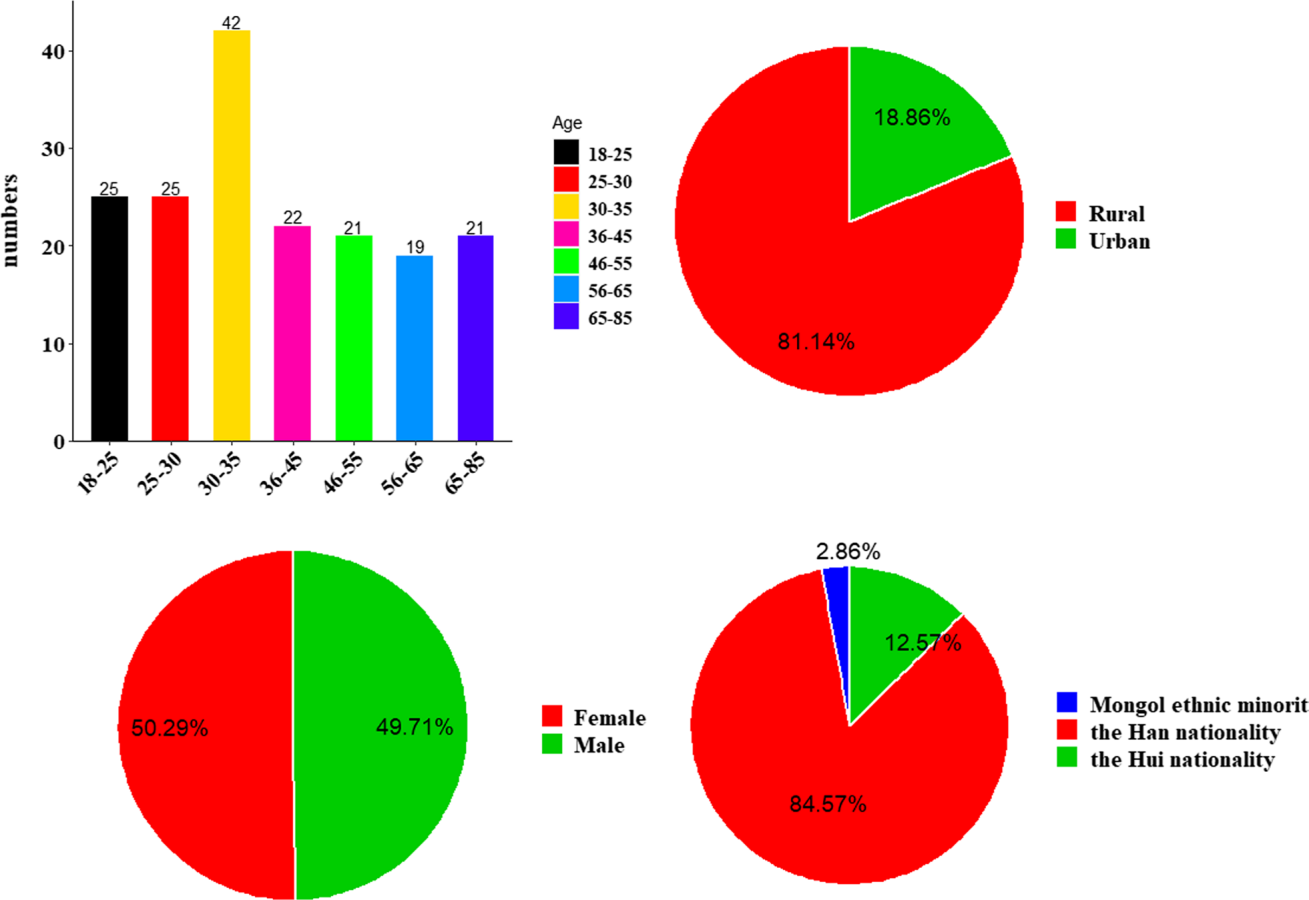
Basic information about the interviewees

The age distribution of 175 interviewees was segmented, and the results showed that all interviewees were between 18 and 85 years of age; 25 were between 18 and 25 years of age, 25 were between 25 and 30 years of age, 42 were between 30 and 35 years of age, 22 were between 36 and 45 years of age, 21 were between 46 and 55 years of age, 19 were between 56 and 65 years of age and 21 were over 65 years of age. Of those interviewed, 142 were of rural origin (i.e., they were born in rural areas and raised in rural areas during childhood and adolescence), accounting for 81.14 percent of all interviewees, and 33 were of urban origin (i.e., they were born and lived in urban areas), accounting for 18.86 percent of the total. There were 87 males and 33 females, with a male-to-female ratio close to 1:1. A total of 148 of the interviewees were of the Han nationality, 22 were of the Hui nationality, and 5 were of the Mongolian nationality (Fig. 2).

The results of the survey showed that the number of species of wild plants eaten by the interviewee was positively correlated with age. Eleven wild plant species were consumed by each of the 25 reporters under 25 years of age, while up to 47 wild plant species were consumed by each of the 21 reporters over 65 years of age, 4.27 times the number of reported species consumed by respondents under 25 years of age. Most of the 11 wild plants eaten by people under the age of 25 are common wild vegetables (with tender stems and leaves), such as *Sonchus wightianus* DC., *Mulgedium tataricum* (L.) DC., *Nostoc commune* Vaucher ex Bornet & Flahault, *Taraxacum mongolicum* Hand.-Mazz., *Allium mongolicum* Regel, the flower of *Robinia pseudoacacia* L., the samara of *Ulmus pumila* L., and the bud of *Toona sinensis* (Juss.) M. Roem.. The number of species consumed by people over 65 years old was significantly higher than the number of species consumed by people in the other age groups, and the difference was mainly due to the consumption of dermatophytes and spermatophytes, such as the bark of *U. pumila* L., the seeds of *Corispermum hyssopifolium* L. and *Chenopodium album* L*.* These plants were eaten only in years with famine. With abundant material on resources and the fact that people are no longer lacking food, these plants are rarely eaten by people.

The consumption of edible wild plant species is also directly related to the growth environment of the interviewee. Individuals from rural areas consumed significantly higher numbers of edible wild plant species than individuals from urban areas. For example, among interviewees between the ages of 30 and 35, 37 wild plants had been consumed by all the interviewees from rural areas, while only 14 wild plants had been consumed by all the interviewees from urban areas. The 14 plants consumed by individuals from urban areas are also mainly wild vegetables, such as *S. wightianus* DC., *N. commune* Vaucher ex Bornet & Flahault, *T. mongolicum* Hand.-Mazz., *A. mongolicum* Regel, and the buds of *T. sinensis* (Juss.) M. Roem.. Furthermore, modern urban food and beverage items are often derived from the species *A. mongolicum* Regel, and the buds of *T. sinensis* (Juss.) M. Roem. have been artificially cultivated.

### Sources of wild edible plants in the Hassan area

A statistical analysis was performed on wild edible plants in the Hassan region. There were 53 species (based on incomplete statistics) of wild edible plants belonging to 24 families in the Hassan region. Compositae and Liliaceae were the most prevalent families, with 8 wild edible plants belonging to Compositae and 7 wild edible plants belonging to Liliaceae, most of which were eaten as wild vegetables. The 8 wild edible plants belonging to Compositae had a relatively wide distribution range and were generally distributed throughout the investigation area; among these 8 plants, *S. wightianus* DC., *M. tataricum* (L.) DC., and *T. mongolicum* Hand.-Mazz. were the most common wild vegetables in the region. The distribution of edible wild plants of Liliaceae is relatively regional, and communities of this species are relatively concentrated. There were four, four, and three species of wild edible plants belonging to *Solanaceae* Juss., *Chenopodiaceae* Vent. and *Rosaceae* Juss., respectively. Most of the wild edible plants belonging to these three families were consumed as snacks.

### Edible parts of the wild edible plants in the Hassan area

**Fig. 3:**
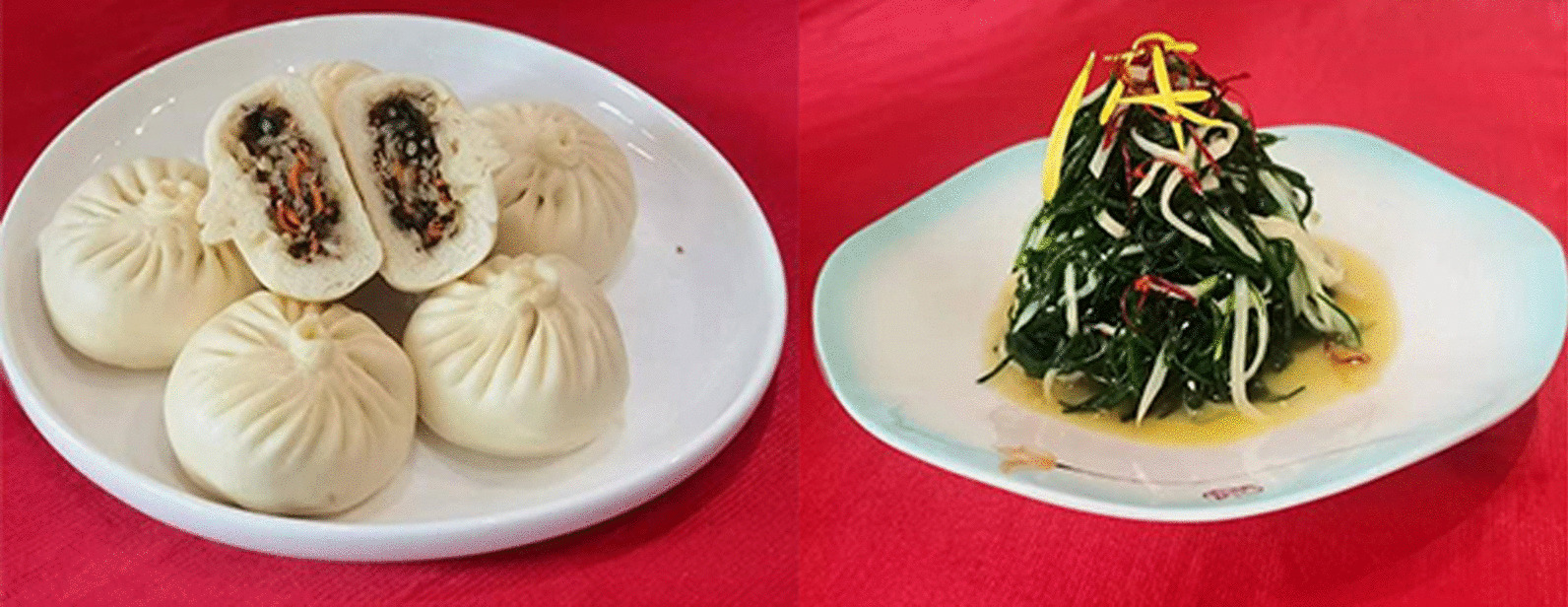
Steamed stuffed bun with ground ear and shallot salad

Among the 53 wild edible plants identified, the tender stems and leaves (including tender buds) were the most commonly used parts of the plants, with 17 species, followed by the fruits (including tender fruits), with 16 species. In addition, the edible plant parts included the roots or rhizomes (bulbs), seeds, and whole plants (Table 1). Plants were consumed in two forms: raw and cooked. Raw food, typically fruit, was mainly used as snacks. Cooked food mainly consisted of vegetables, and some wild edible plants have multiple edible parts or multiple forms of consumption (Fig. 3). In addition, there are plants that are mainly used as flavoring and in medicinal diets, such as *Thymus quinquecostatus* Celak., *Cistanche deserticola* Ma, and *Cynomorium songaricum* Rupr.. The edible parts of herbs mainly consisted of young stems and leaves, which were typically eaten as vegetables, while tall woody plants mainly provided fresh and tender fruits or mature fruit seeds, which were typically eaten as snacks. The wild edible plants whose edible parts were tender stems and leaves and tender buds were generally eaten as vegetables after being mixed with cold elements or pickled, and the fruits, roots, and rhizomes were mainly eaten fresh as snacks. The seeds and skins of fruits were typically used as an emergency resource and supplementary food in times of famine. Currently, given the abundance of resources, seeds, such as those of *C. hyssopifolium* L. and *C. album* L., are rarely eaten by people*.*

**Table 1:** Catalogue of wild edible plants in mixed area of Hui and Han in Northwest China

Families and genera	Plant name	Local name in Chinese	Local name in pinyin	Plant type	Edible part	Food category	Edible method	Voucher numbers
*Asteraceae* Bercht. & J.Presl	*Mulgedium tataricum* (L.) DC.	麻苦菜	Makucai	Perennial herb	Tender leaf / rhizome	Vegetables, medicinal materials	Cold and dressed with sauce /pickle	GSBY-2018-L-003
	*Sonchus wightianus* DC.	甜苦菜	Tiankucai	Perennial herb	Tender leaf / rhizome	Vegetables	Cold and dressed with sauce /pickle	GSBY-2018-L-004
	*Taraxacum mongolicum* Hand.-Mazz.	黄黄菜/黄儿菜	Huanghuangcai/Huangercai	Perennial herb	Tender stem and leaf/Roots	Vegetables/snacks/medicinal materials	Fresh/cold and dressed with sauce /pickle	GSBY-2018-L-014
	*Cirsium souliei* (Franch.) Mattf. ex Rehder & Kobuski	刺甲盖	Cijiagai	Perennial herb	Root	Snack	Fresh food	GSBY-2018-L-007
	*Cichorium intybus* L.	小苦菜	Xiaokucai	Perennial herb	Tender leaf / rhizome	Vegetables	Fresh/cold/pickled	GSBY-2018-L-011
	*Tragopogon pratensis* L.	羊奶子	Yangnaizi	Perennial herb	Bud	Snack	Fresh food	GSBY-2018-L-021
	*Artemisia annua* L.	黄蒿	Huanghao	Annual herb	Bud	Vegetables		GSBY-2018-L-016
	*Artemisia lavandulaefolia* DC.	艾蒿	Aihao	Perennial herb	Bud	Auxiliary food	Steam cooked food with mixed flour (moxa bun)	GSBY-2018-L-020
*Rosaceae* Juss	*Rosa omeiensis* Rolfe	油瓶瓶	Youpingping	Large shrub	Ripe fruit	Snack	Fresh food after fresh frost	GSBY-2018-L-077
	*Fragaria vesca* L.	莓子	Meizi	Perennial herb	Ripe fruit	Snack	Fresh food	GSBY-2018-L-053
	*Prunus armeniaca* var. ansu Maxim.	野杏子	Yexingzi	Arbor	Ripe fruit	Snack	Fresh food	GSBY-2018-L-045
*Amaryllidaceae* J.St.-Hil	*Allium schoenoprasum* L.	沙葱	Shacong	Perennial herb	Tender leaf	Vegetables	Cold and dressed with sauce /Steamed buns	GSBY-2018-L-036
	*Allium strictum* Schrad.	小蒜	Xiaosuan	Perennial herb	Bulb	Snacks/vegetables	Fresh food	GSBY-2018-L-033
	*Allium przewalskianum* Regel	石蒜	Shisuan	Perennial herb	Bulb	Snacks/vegetables	Fresh food	GSBY-2018-L-034
	*Allium mongolicum* Regel	扁韭菜	Bianjiucai	Perennial herb	Tender leaf	Vegetables	Cold and dressed with sauce /pickle	GSBY-2018-L-074
	*Allium bidentatum* Fisch. ex Prokh. & Ikonn.-Gal.	野韭菜	Yejiucai	Perennial herb	Tender leaf	Vegetables	Cold and dressed with sauce /pickle	GSBY-2018-L-052
	*Lilium pumilum* Redouté	山丹丹	Shandandan	Perennial herb	Bulb/flower	Vegetables	Bulb salad/flower soup	GSBY-2018-L-102(B)
*Asphodelaceae* Juss	*Hemerocallis citrina* Baroni	黄花菜	Huanghuacai	Perennial herb	Bud	Vegetables	Cold and dressed with sauce /soup	GSBY-2018-L-009
*Fabaceae* Lindl	*Medicago sativa* L.	苜蓿芽子	Muxuyazi	Perennial herb	Bud	Vegetables	Cold and dressed with sauce	GSBY-2018-L-029
	*Robinia pseudoacacia* L.	槐花	Huaihua	Arbor	Bud/flower	Snack	Fresh/cooked food	GSBY-2018-L-005
*Solanaceae* Juss	*Lycium chinense* Mill.	枸杞	Gouqi	Large shrub	Fruit	Snack	Fresh/ dried tea/ soup (herbal)	GSBY-2018-L-010
	*Lycium ruthenicum* Murray	黑枸杞	Heigouqi	Small shrub	Fruit	Snack	Fresh/ dried tea/ soup	GSBY-2018-L-066
	*Solanum septemlobum* Bunge	野西红柿	Yexihongshi	Annual herb	Fruit	Snack	Fresh food	GSBY-2018-L-083
	*Solanum tuberosum* L.	酸楸子	Suanqiuzi	Annual herb	Fruit	Snack	Fresh food after frost	GSBY-2018-L-018
*Brassicaceae* Burnett	*Lepidium apetalum* Willd.	辣辣	Lala	Annual herb	Tender root	Snack	Fresh food	GSBY-2018-L-027
	*Isatis indigotica* Fortune ex Lindl.	大青叶子	Daqingyezi	Annual herb	Tender leaf	Vegetables	Cold and dressed with sauce	GSBY-2018-L-048
	*Brassica campestris* L.	芸薹	Yuntai	Annual herb	Tender stem and leaf/Seeds	Vegetable/oil	Cold and dressed with sauce/fried food/oil from its seeds	GSBY-2018-L-112
*Amaranthaceae* Juss	*Chenopodium album* L.	灰条	Huitiao	Annual herb	Bud/Seed	Vegetables, supplementary foods	Steamed buns with tender bud/ Cold and dressed with sauce/making steamed bread with seed powder	GSBY-2018-L-009
	*Kochia scoparia* (L.) Schrad.	芡扫帚	Qiansaozhu	Annual herb	Bud	Vegetables	Cold and dressed with sauce	GSBY-2018-L-017
	*Corispermum hyssopifolium* L.	绵蓬	Mianpeng	Annual herb	Ripe seed	Auxiliary food	Making steamed bread with seed powder	GSBY-2018-L-055
	*Halogeton glomeratus* (M.Bieb.) Ledeb.	水蓬	Shuipeng	Annual herb	Whole grass	Condiment	Burn into ash to make edible alkali	GSBY-2018-L-056
*Elaeagnaceae* Juss	*Hippophae rhamnoides* L.	沙棘子	Shajizi	Large shrub	Fruit/Leaf	Snacks/tea	Fresh fruit/leaf tea	GSBY-2018-L-062
	*Elaeagnus angustifolia* L.	沙枣	Shazao	Arbor	Ripe fruit	Snack	Fresh after removing astringency after frost	GSBY-2018-L-010
*Berberidaceae* Juss	*Berberis diaphana* Maxim.	酸酸/酸溜溜	Suansuan/Suanliuliu	Small shrub	Ripe fruit/ tender leaf	Snack	Fresh food	GSBY-2018-L-047
*Apocynaceae* Juss	*Cynanchum thesioides* (Freyn) K.Schum.	蒿果/蒿瓜	Haoguo/Haogua	Perennial herb	Tender fruit	Snack	Fresh food	GSBY-2018-L-094
	*Cynanchum chinense* R.Br.	羊奶角	Yangnaijjiao	Perennial herb	Fruit	Snack	Fresh food	GSBY-2018-L-091
*Geraniaceae* Juss	*Geranium wilfordii* Maxim.	罗棠苗	Luotangmiao	Annual herb	Tender root	Snack	Fresh food	GSBY-2018-L-093
*Iridaceae* Juss	*Iris tenuifolia* Pall.	梭瓜	Suogua	Perennial herb	Tender fruit	Snack	Fresh food	GSBY-2018-L-098
*Lamiaceae* Martinov	*Thymus quinquecostatus* Celak.	地椒椒/百里香	Dijiaojiao/Bailixiang	Perennial herb	Aboveground part	Condiment	After drying and crushing, mix it into the fried flour	GSBY-2018-L-057
	*Stachys sieboldii* Miq	地瓜/螺丝菜	Digua/Luosicai	Annual herb	Root tuber	Vegetables	Cold and dressed with sauce /pickle	GSBY-2018-L-125
*Ulmaceae* Mirb	*Ulmus pumila* L.	榆钱	Yuqian	Arbor	Fruit/skin	Snacks/supplements	Fresh /steamed food	GSBY-2018-L-068
*Salicaceae* Mirb	*Populus davidiana* Dode	白杨树	Baiyangshu	Arbor	Tender bud/skin	Vegetables	Cold and dressed with sauce /cooking	GSBY-2018-L-069
*Orobanchaceae* Vent	*Cistanche deserticola* Ma	肉苁蓉	Roucongrong	Annual herb	Complete stool	Health foods	Medicated diet	GSBY-2018-P-002
*Cynomoriaceae* Endl. ex Lindl	*Cynomorium songaricum* Rupr.	锁阳	Suoyang	Annual herb	Complete stool	Health foods	Medicated diet	GSBY-2018-P-003
*Meliaceae* Juss	*Toona sinensis* (Juss.) M.Roem.	香椿	Xiangchun	Arbor	Bud	Vegetables	Cold and dressed with sauce	GSBY-2018-L-070
*Apiaceae* Lindl	*Daucus carota* L.	野胡萝卜	Yehuluobo	Annual herb	Root	Snack	Cooked for consumption	GSBY-2018-L-013
*Rhamnaceae* Juss	*Ziziphus jujuba* Mill.	酸枣	Suanzao	Large shrub	Fruit	Snack	Fresh/sun-dried food	GSBY-2018-L-125
*Polygonaceae* Juss	*Rheum nanum* Siev. ex Pall.	黄狗卵子	Huanggouluanzi	Perennial herb	Root	Snack	Roasted cooked food (similar to potatoes and sweet potatoes)	GSBY-2018-P-011
*Poaceae* Barnhart	*Avena fatua* L.	燕麦	Yanmai	Annual herb	Seed	Auxiliary food	To make fried flour /fermented glutinous rice	GSBY-2018-L-024
	*Panicum miliaceum* L.	火穗	Huosui	Annual herb	Ears of tender diseased plants	Snack	Fresh food	GSBY-2018-L-026
*Zygophyllaceae* R.Br	*Zygophyllum fabago* L.	面蛋蛋	Miandandan	Small shrub	Fruit	Snack	Fresh food	GSBY-2018-L-088(B)
*Nostocaceae*	*Nostoc commune* Vaucher ex Bornet & Flahault	地软子/地达菜	Diruanzi/Didacai	Fungus	Complete stool	Auxiliary food	Steamed stuffed bun	GSBY-2018-P-013
	*Nostoc flagelliforme* Born. et Flah.	头发菜	Toufacai	Fungus	Complete stool	Auxiliary food	Omelette	GSBY-2018-P-014

Nfried noodles is a kind of food (dry food) made in the north by parching and grinding grains such as wheat and oats into flour

### Quantitative evaluation of edible wild plants in the Hassan area

**Fig. 4:**
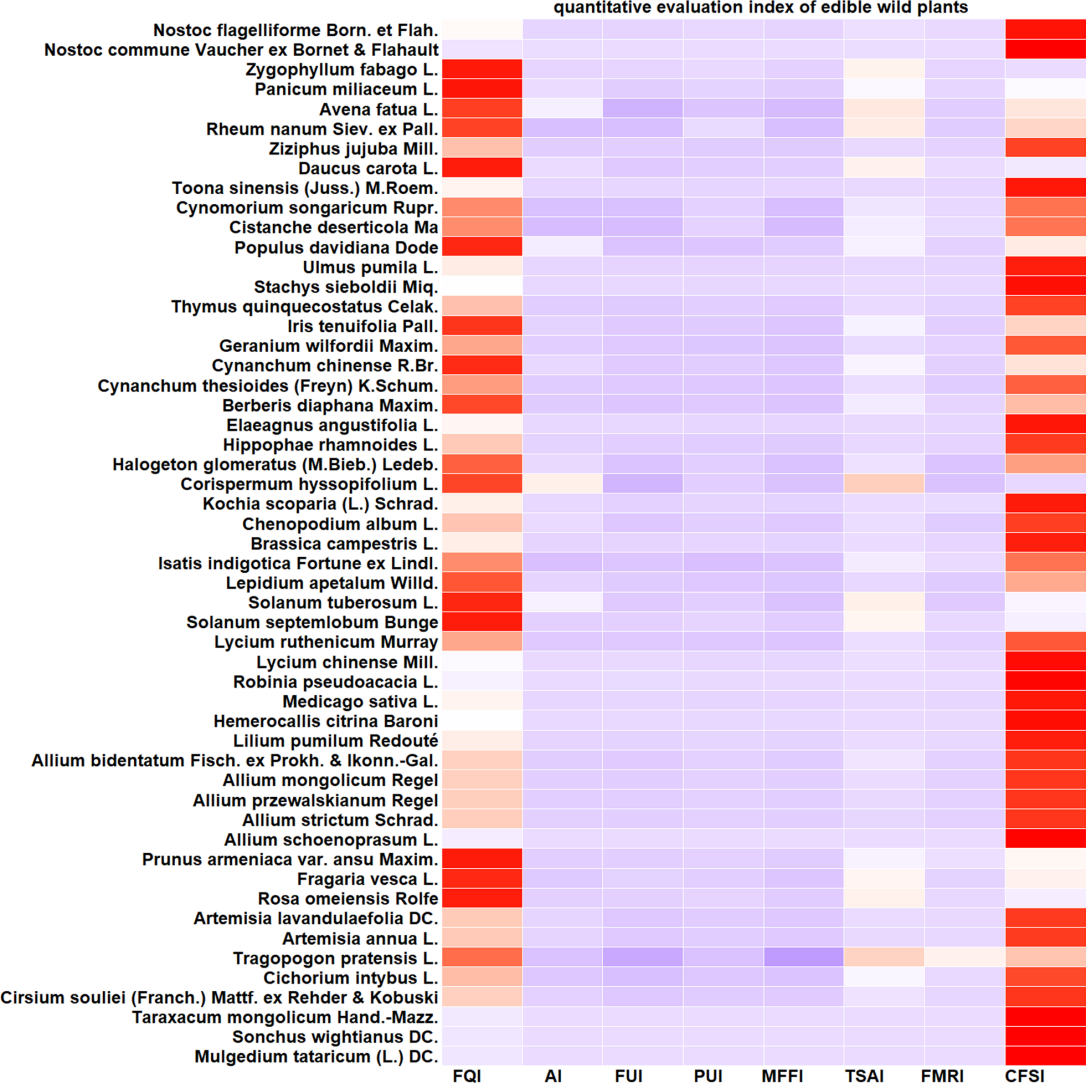
Heatmap of edible wild plants in the Hassan area

The results of the comparison of the CFSI values of wild edible plants near Mount Hassan are shown in Table 2 and Fig. 4. The edible wild plants in the region were clustered based on their CFSI values to screen out the wild edible plants that were widely used in the region (i.e., those with high values) and played an important role in the traditional diet of local people. The plants with the highest importance values (CFSI > 500) are *M. tataricum* (L.) DC., *N. commune* Vaucher ex Bornet & Flahault, *S. wightianus* DC., *T. mongolicum* Hand.-Mazz., *Allium schoenoprasum* L., *Hemerocallis citrina* Baroni, *Elaeagnus angustifolia* L., *Medicago sativa* L., *U. pumila* L., *Stachys sieboldii* Miq., and *T. sinensis* (Juss.) M. Roem., consisting mainly of wild vegetables. These group of wild edible plants play an important role in the lives of local people; for example, *M. tataricum* (L.) DC., *S. wightianus* DC., *A. schoenoprasum* L., *M. sativa* L., and *N. commune* Vaucher ex Bornet & Flahault have been used in dishes prepared by local people since ancient times. Prior to the widespread use of greenhouses, *S. wightianus* DC., *A. schoenoprasum* L., and *M. sativa* L. were the main vegetables consumed by local people in spring. Currently, these vegetables are the most representative wild vegetable varieties for the catering organizations in the region. The plants with the second highest importance values (500 > CFSI ≥ 100) included mainly wild vegetables and plants used as snacks, such as *Z. jujuba* Mill. and *Hippophae rhamnoides* L., and these edible wild plants also had good development value. The reason for their lower CFSI values was mainly related to their narrow distribution range and limited edible parts. Most plants with the third highest importance values (100 > CFSI ≥ 10) were consumed typically as snacks, and some of them had high medicinal value, such as *C. songaricum* Rupr., *Isatis indigotica* Fortune ex Lindl., and *C. deserticola* Ma. The plants with the fourth highest importance values (10 > CFSI) were mainly distributed in only certain areas or had poor taste; these plants were mostly collected and eaten in times of famine. The interviewees who reported eating these species were mainly elderly individuals, and currently the species in this group, such as *C. hyssopifolium* L. and *Populus davidiana* Dode, are rarely eaten by people.

**Table 2:** Quantitative evaluation index of edible wild plants in Hassan area

Plant name	FQI	AI	FUI	PUI	MFFI	TSAI	FMRI	CFSI
*M. tataricum*	165	4.0	4.0	3.00	1.50	7.5	5.0	4455.00
*S. wightianus*	158	4.0	4.0	3.00	1.50	7.5	5.0	4266.00
*T. Mongolicum*	162	4.0	3.0	3.00	1.50	7.5	5.0	3280.50
*C. souliei*	24	3.0	1.0	2.00	1.50	6.5	4.0	56.16
*C. intybus*	18	2.0	1.0	2.00	1.50	7.5	4.0	32.40
*T. Pratensis*	11	2.0	1.0	2.00	0.50	6.5	5.0	7.15
*A. Annua*	34	4.0	1.0	2.00	1.00	5.5	5.0	74.80
*A. lavandulaefolia*	28	4.0	1.0	2.00	1.00	5.5	5.0	61.60
*R. omeiensis*	36	1.0	1.0	1.50	0.50	9.0	2.0	4.86
*F. vesca*	35	1.0	2.0	1.50	0.50	9.0	2.0	9.45
*P. armeniaca*	52	1.0	1.0	1.50	0.50	7.5	4.0	11.70
*A. schoenoprasum*	137	3.0	4.0	3.00	1.50	9.0	3.0	1997.46
*A. strictum*	111	2.0	1.0	3.00	1.50	9.0	3.0	269.73
*A. przewalskianum*	76	2.0	1.0	3.00	1.50	9.0	3.0	184.68
*A. mongolicum*	58	2.0	1.0	3.00	1.50	9.0	3.0	140.94
*A.bidentatum*	34	2.0	1.0	3.00	1.50	9.0	3.0	82.62
*L. pumilum*	63	2.0	1.0	3.00	1.50	9.0	5.0	255.15
*H. citrina*	137	3.0	3.0	1.50	1.50	9.0	4.0	998.73
*M. sativa*	121	4.0	4.0	0.75	1.50	9.0	3.0	588.06
*R. pseudoacacia*	89	5.0	3.0	1.50	1.00	10.0	5.0	1001.25
*L. chinense*	45	5.0	4.0	1.50	0.50	10.0	5.0	337.50
*L. ruthenicum*	63	2.0	2.0	1.50	0.50	10.0	5.0	94.50
*S. septemlobum*	38	1.0	1.0	1.50	0.50	9.0	2.0	5.13
*S. tuberosum*	22	4.0	1.0	1.50	0.50	6.5	1.0	4.29
*L. apetalum*	86	5.0	2.0	1.00	0.50	6.5	2.0	55.90
*I. indigotica*	31	1.0	2.0	1.00	1.50	7.5	5.0	34.88
*B. campestris*	42	2.0	2.0	3.00	1.50	7.5	3.0	170.10
*C. album*	27	5.0	1.0	2.50	1.50	5.5	2.0	55.69
*K. scoparia*	29	4.0	1.0	2.50	1.50	5.5	5.0	119.63
*C. hyssopifolium*	11	4.0	0.5	1.50	1.00	5.5	1.0	1.82
*H. glomeratus*	45	5.0	1.0	3.00	0.75	6.5	1.0	32.91
*H. rhamnoides*	62	4.0	2.0	1.50	0.50	7.5	5.0	139.50
*E. angustifolia*	136	5.0	5.0	1.50	0.50	9.0	3.0	688.50
*B. diaphana*	58	2.0	1.0	1.50	0.50	9.0	4.0	31.32
*C. thesioides*	72	3.0	2.0	1.50	0.50	10.0	3.0	97.20
*C. chinense*	44	3.0	1.0	1.50	0.50	7.5	2.0	14.85
*G. wilfordii*	56	4.0	2.0	1.00	0.50	7.5	5.0	84.00
*I. tenuifolia*	54	3.0	1.0	1.50	0.50	9.0	2.0	21.87
*T. quinquecostatus*	66	2.0	1.0	3.00	0.75	9.0	5.0	133.65
*S. sieboldii*	78	4.0	3.0	1.00	1.50	9.0	4.0	505.44
*U. pumila*	129	5.0	2.0	1.50	1.00	9.0	3.0	522.45
*P. davidiana*	32	5.0	0.5	0.75	1.50	5.5	2.0	9.90
*C. deserticola*	29	1.0	1.0	4.00	0.75	7.5	5.0	32.63
*C. songaricum*	36	1.0	1.0	4.00	0.75	7.5	5.0	40.50
*T. sinensis*	121	4.0	4.0	0.75	1.50	9.0	3.0	588.06
*D. carota*	25	2.0	0.5	1.00	1.00	6.5	2.0	3.25
*Z. jujuba*	78	2.0	3.0	1.50	0.50	9.0	5.0	157.95
*R. nanum*	21	1.0	1.0	3.00	1.00	7.5	2.0	9.45
*A. fatua*	17	4.0	0.5	1.50	1.00	6.5	2.0	6.63
*P. miliaceum*	46	3.0	0.5	1.50	0.50	7.5	2.0	7.76
*Z. fabago*	32	1.0	1.0	1.50	0.50	7.5	1.0	1.80
*N. commune*	162	5.0	4.0	4.00	1.50	9.0	4.0	6998.40
*N. flagelliforme*	37	2.0	1.0	4.00	1.50	9.0	5.0	199.80

FQI—frequency index, AI—commonness index, FUI—frequency index, PUI—utilization site index, MFFI—multifunctional food utilization index, TSAI—taste evaluation index, and FMRI—medicinal diet function index

## Discussion

The wild edible healthy plants collected in this investigation have obvious regional characteristics. Except for a few plants that are widely distributed in China and eaten as wild vegetables in most areas, such as *S. wightianus* DC., *C. album* L*.*, and *T. mongolicum* Hand.-Mazz., the vast majority of plants are typical drought-tolerant plants, and *C. deserticola* Ma, *C. songaricum* Rupr., *N. flagelliforme* Born. et Flah., *Z. fabago* L., *R. nanum* Siev. ex Pall. are the representative plants in arid areas or desert areas. We also found that some plants were eaten and widely distributed in other areas of Gansu but were not in the investigated area; for example, *Plantago asiatica* L. and *Urtica fissa* E.Pritz. were eaten in Zhouqu, Gansu [31]. This indicated that there were differences in the understanding of wild edible healthy plant resources among regions.

Although the survey area is inhabited by multiple ethnic groups, such as the Han, Hui, and Mongolian groups, almost all ethnic groups have basically Sinicized, and no significant difference has been found in the knowledge of wild edible healthy plants among the groups. The use of local names for the collected plants makes it difficult to determine their extent nationally; however, these names often have a direct relationship with the taste, edible parts, and categories of the plants. For example, Kucai(*M. tataricum* (L.) DC., *S. wightianus* DC.), Suanliuliu(*B. diaphana* Maxim*.*), Lala(*L. apetalum* Willd.), Suanqiuzi(*S. tuberosum* L.) are named for their taste, and Youpingping(*R. omeiensis* Rolfe), Yangnaizi(*T. pratensis* L.), Huanggouluanzi(*R. nanum* Siev. ex Pall.) are named for their shapes. Youpingping refers to the similarity of the food resource to a bottle of oil, Yangnaizi is named for its resemblance to sheep nipples, and Huanggouluanzi is yellow and shape like a dog's testicles, while Toufacai(*N. flagelliforme* Born. et Flah.) is a kind of edible vegetable with an appearance like hair. Plants from a wide range of edible groups, such as vegetables, often contain “cai” (Chinese pronunciation) in their local names, which suggests that researchers have paid attention to this unique phenomenon [32]. In addition, onion and garlic are traditionally used for flavoring in China, and wild edible onion and garlic have similar functions, being used for flavoring, with the plant forms (or species) being close to the traditionally planted onions and garlic.

Knowledge about edible wild plants in the survey area gradually decreased with time (calculated based on the age of the interviewees). This result may be directly related to the social and economic development in China. In years of scarcity, wild edible plants are not only a supplement to daily food but also life-saving food and medicine in many cases. Currently, except a few wild edible plants that can be used as tonics, many of these plants, such as *C. deserticola* Ma, *C. songaricum* Rupr., and *N. flagelliforme* Born. et Flah., will be collected and stored for consumption in other seasons.

Another finding that needs to be explained is that there are certain kinds of mushrooms in this area (the local people refer to similar fungi collectively as mushrooms), but they are rarely eaten. The reason for their lack of consumption is that local people think that mushrooms are prone to attracting maggots, which represent unclean conditions. This may be the reason why local people seldom eat mushrooms, which differs completely from the understanding of the edibility and value of mushrooms in other areas of Gansu [33]. Of course, there have been no incidences of mushroom poisoning in this area. In addition, the residents of this area also have knowledge about other poisonous plants. The roots of *Aconitum* L. and the *Anisodus Link* et Otto are not edible. A local legend states that a shepherd and a young child died because they accidentally ate *Aconitum szechenyianum* Gáyer and the roots of *Anisodus Link* et Otto. These two plants are explicitly listed in the local list of plants not suitable for consumption, and this knowledge has been passed down from generation to generation, in contrast to the habit of eating *Aconitum carmichaeli* Debx. in other parts of China [34].

With the development of the economy, there has been a gradual increase in the desire for wild edible plant resources among consumers. Developing tourism is an important means of economic development in western China [35]. Wild and healthy natural vegetables have become an important publicity point for attracting tourists; this publicity causes consumers to have a “curiosity” mentality about wild edible plants, and they regard the consumption of these plants as a fashionable behavior [36]. Due to these psychological and behavioral factors, the demand for wild edible plant resources has greatly increased. However, the study region is in the arid area of the Loess Plateau in northern Gansu Province, and its ecological environment is extremely fragile. A large number of wild edible plants are collected in this area, especially wild plants with edible parts that include roots, rhizomes, and whole plants. In the investigated area, *C. deserticola* Ma, *C. songaricum* Rupr., and *N. flagelliforme* Born. et Flah. are often targeted for collection.

The total number of wild edible and healthy plant resources collected in the study area is small, mainly due to the low species richness in the area caused by the ecological environment. Nevertheless, the extent of exploitation of edible plants and plant resources throughout the region is actually quite high. There were no differences in the information provided by consumers from the Han, Hui, and Mongolian groups about the healthy wild edible plants collected. The species investigated included mainly herbaceous plants, which were mostly consumed as vegetables, while a small number of woody plants provided snacks, and mushrooms (fungi) were generally ignored. Wild plant resources are used by residents in this region for medicine and food as well as the production of various tools (such as basket weaving with *Achnatherum splendens* (Trin.) Nevski and broom binding with *Achnatherum inebrians* (Hance) Keng). Some plants can be used for multiple purposes, although this pattern requires further investigation.

## Data Availability

All data, materials, and information are collected from the study sites.
